# Investigation on the occupational situation of clinical research associates in China

**DOI:** 10.3389/fmed.2025.1499237

**Published:** 2025-01-23

**Authors:** Can Du, Qi Han, Ziqian Lu, Jia Li

**Affiliations:** National Clinical Research Center for Oral Diseases, Clinical Research Department, West China Hospital of Stomatology, Sichuan University, Chengdu, Sichuan, China

**Keywords:** clinical research associate, clinical trial, occupational situation, monitoring, institution, research site

## Abstract

Clinical research associates (CRAs) are the sponsor’s representatives, monitor the process and quality of clinical trials, their professional quality can affect the quality of clinical trials. But there is a conflict between the large number of CRAs personnel and unsatisfactory quality of clinical trial monitoring, and the reason is unknown. Therefore, our study investigated the occupational status of CRAs through a questionnaire survey. A total of 401 eligible questionnaires were included in the final analysis. Of whom, 71.32% were females, the average age is 27.86 years (range: 22–46 years), 95.01% had a bachelor’s degree or above, and 60% had a pharmaceutical major. 76.31% had month income of <20,000 Yuan RMB, and dissatisfied with their current salary level. Over 80% weekly working time more than 40 h. 79.3% CRAs traveling for less than 7 days per month under the impact of the COVID-19 epidemic. 80% of them are satisfied with promotion channels and company training. Furthermore, Through the analysis of satisfaction differences, we found that CRAs with different educational backgrounds have significant differences in career satisfaction. It was suggested by more than 80% CRAs that the application materials for clinical trials should be unified nationwide and an ethical mutual recognition system. In conclusion, our study is the first large-scale survey of CRAs in China, the current professional situation of CRAs is considerable, but overall it still needs further improvement and enhancement, the monitoring difficulties faced by CRAs mainly come from hospitals, companies, and themselves. With the robust growth of clinical trials in China, hospitals and companies need to improve the professional quality and status of CRAs through a series of measures, further driving the improvement of clinical trial quality.

## 1 Introduction

In recent years, an increasing number of people embark on their careers related to clinical trials as various clinical trials are launched in China. Clinical research associates (CRAs), are the practitioners who go to institutions as the representatives of the sponsor to monitor the process and quality of clinical trials (1), guarantee the rights and interests of subjects and verify the accuracy of trial data in order to ensure that clinical trials follow the established protocol and meet the requirements of Good Clinical Practice (GCP) and other corresponding laws and regulations (2). In other words, CRAs play their roles in monitoring and guaranteeing the quality of clinical trials. As the clinical trial industry gains momentum, an increasing number of people choose to serve as CRAs (3). As research shows, China and the United States each have nearly 30,000 CRAs respectively, whose professional quality can also affect the quality of clinical trials (4, 5).

The responsibilities a CRA assumes are defined both in The International Council for Harmonization of Technical Requirements for Pharmaceuticals for Human Use (ICH) and China’s own GCP, but either unified access standards for this industry or corresponding industry norms are lacking in China at present. The current basic requirement for one to be a CRA is only to equip himself or herself with certain medical or pharmaceutical knowledge and receive GCP training. However, the yearly inspection results of clinical trial projects published by the National Medical Products Administration (NMPA) and its provincial branches reveal many problems related to monitoring, such as frequent replacement of CRAs, CRAs’ unfamiliarity with clinical trial protocols, inconsistency between monitoring reports and factual conditions, and weak feasibility of monitoring schemes (6–8).

The conflict between the large number of practitioners and the unsatisfactory monitoring quality necessitates a relevant research to analyze the performance of CRAs so as to find out the difficulties in monitoring and improve monitoring quality in a targeted manner. But it is unfortunate that, after consulting a large number of literatures, our research group failed to find many articles on the systematic investigation into CRAs at present. On this basis, we initiated this research into the background, workloads and difficulties of CRAs.

## 2 Methods

### 2.1 Survey design and survey content

The survey questionnaire is released through Wenjuanxing,^1^ inviting CRAs across the country to fill out the questionnaire. The calculated sample size yields 271 individuals by the Formula. Taking into account a dropout rate of 20%, the minimum sample size required for the study is adjusted to 339 individuals to maintain statistical power and account for potential attrition. The research period is 2 months, all survey questionnaires are forwarded and submitted on WeChat mobile or computer platforms.


$$\mathrm{Formula}:n=\frac{z^{2}*p*\left(1-p\right)}{e^{2}}$$

In the context of statistical sampling, (*n*) denotes the sample size, (*z*) represents the z-score associated with the desired level of confidence, (*p*) signifies the sample proportion expressed as a decimal, and (*e*) indicates the margin of error also expressed as a decimal. When estimating the sample size with Power (1 − *β*) = 0.9, corresponding to a *z*-score of 1.645, and a margin of error (e) of 5%, assuming the worst-case scenario where the proportion (*p*) is 0.5 (i.e., 50% of individuals select a particular option), the calculated sample size yields 271 individuals. Taking into account a dropout rate of 20%, the minimum sample size required for the study is adjusted to 339 individuals to maintain statistical power and account for potential attrition.

The investigation was carried out in the form of a questionnaire which contained five sections. The first part represented the basic information collection of CRAs, including demographic information, educational background, length of service, employer background, salary, etc., which gave a preliminary introduction to the current structure of CRAs. The second pertained to the collection of work, including workloads, travel frequency, and training, etc. The third concerned CRAs’ satisfaction with institutions, in which the work performance of management departments, ethics committees and investigators were scored by CRAs. The fourth was related to career satisfaction in such aspects as salary, promotion channels and internal training of the companies. The fifth was relevant to open questions, in which the CRAs were asked to put forward a wide range of suggestions for the industry.

### 2.2 Data processing and analysis

After the research period ends, terminate the questionnaire operation. Download the backend data in the option text format and save it in Excel format. Double check the data and input it. Exclusion criteria for questionnaire data: (1) There are obvious logical or data errors in the answers; (2) All the answers were similar.

In this study, all statistical analyses and graphing were performed using IBM SPSS 25.0 and GraphPad Prism 8. The general characteristics of the study population were analyzed using descriptive statistical methods. For continuous variables, we used the mean ± standard deviation for representation and one-way ANOVA to compare satisfaction scores across different groups. All statistical tests were conducted as two-tailed, with the significance level set at 0.05.

### 2.3 Ethics

The study was conducted in accordance with the Declaration of Helsinki, and approved by the Ethics Committee of West China Hospital of Stomatology Sichuan University (WCHSIRB-D-2021-550). Participants voluntarily click on the link to fill out the questionnaire. Before filling out the questionnaire, they have been informed of the research purpose and informed that “submitting answers” is considered informed consent. Participants can exit at any time during the questionnaire filling process.

## 3 Results

### 3.1 Basic information

After the 409 questionnaires collected within 2 months were examined, and 401 questionnaires were verified to be valid, with a validity rate of approximately 98.04%. Of the 401 CRAs from 59 cities of 26 provincial-level administrative regions in China, the average age was 27.86 (22–46); 71.32% were women, and 95.01% held bachelor’s degrees or above, with most majoring in pharmacy, nursing and medicine, and the rest in engineering, chemistry and management, etc. Compared with CRAs in other countries, the gender and educational background of practitioners are similar, mostly female with a bachelor’s degree or above (6). When it came to previous work experience, 37.41% worked as CRAs upon graduation, 26.18% changed their careers to this occupation from clinical research coordinators (CRCs), 19.95% from R&D or sales positions in enterprises, 10.47% from hospitals as nurses, medical technicians and doctors, etc., and the rest from non-medical related positions, such as teachers, salesmen and animal experimenters. In the category of clinical trials, 74.31% were engaged in drug clinical trials while 23.19% were devoted to medical device clinical trials, and the rest were dedicated to *in vitro* diagnostics (IVDs), vaccines and scientific research projects. As for the types of employers, 59.10% of CRAs belonged to contract research organizations (CROs) and the rest came from sponsors. In terms of position level and compensation, 43.14% of the respondents worked as primary CRAs, and 56.11% earned 100,000–200,000 RMB per year. The annual income of Canada’s CRA is $40,000–$70,000 (6), according to the PPP (Purchasing Power Parity) in Canada and China, it’s indicated that the income have no difference between two countries (9). See Table 1 for details.

**Table 1:** The basic information of CRAs.

Classification	Number (N)	Proportion (%)
Gender		
Male	115	28.68
Female	286	71.32
Age		
21–25	93	23.19
26–30	237	59.10
31–35	61	15.21
36–40	8	2.00
>40	2	0.50
Educational backgrounds		
Below Junior College	2	0.50
Junior college	18	4.49
Undergraduate	316	78.80
Master	64	15.96
Doctor	1	0.25
Majors		
Pharmacy	240	59.85
Nursing	70	17.46
Medicine	43	10.72
Biology	20	4.99
Others	28	6.98
Previous work		
Working as CRAs upon graduation	150	37.41
CRC	105	26.18
In pharmaceutical Enterprises	80	19.95
In hospitals	42	10.47
Statistics	2	0.50
Others	22	5.49
Categories of clinical trials		
Drugs	298	74.31
Medical devices	93	23.19
IVDs	2	0.50
Vaccine	1	0.25
Others	7	1.75
Types of Enterprises		
CRO	237	59.10
Sponsor	164	40.90
Title Rank		
Primary CRAs	173	43.14
Intermediate CRAs	113	28.18
Advanced CRAs	63	15.71
Project manager	48	11.97
Project director	4	1.00
Annual Income (RMB)		
Below 100,000	81	20.20
100,000–200,000	225	56.11
200,000–300,000	70	17.46
300,000–500,000	23	5.73
Above 500,000	2	0.50

According to the survey results, the respondents, with an average length of employment of 2.80 years, had worked for 1.76 companies on average—that is, they had worked for each company for about 1.59 years. This group of data reflected the high turnover of this occupation (3, 6). The questionnaire asked the respondents to fill in the numbers of both the projects they had accomplished completely from project establishment to completement and those they had taken over halfway. The results showed that the former number averaged out at 2.13 while the latter at 10.12. One of the reasons why the latter was nearly five times as large as the former may be frequent job switch.

In terms of the types of CROs, foreign-funded enterprises accounted for 18.56%, Chinese enterprises for 57.81%, and joint ventures for 23.63%. On the part of sponsors, the proportions for foreign enterprises, Chinese enterprises and joint ventures were 15.24, 57.93, and 26.83%, respectively. Similar to sponsors, Chinese-funded CROs took up the largest proportion of enterprise types, demonstrating that currently the majority of clinical trials were carried out by Chinese local enterprises in China, and most of the trials were undertaken by professional CROs (10).

In respect of educational background, nearly 60% of the CRAs were from pharmacy majors, and 77.33% (116 people) of those who joined CRAs upon graduation were from pharmacy majors, showing that the CRA occupation has become quite attractive to pharmaceutical students. The fact that about a quarter of CRAs switched their occupations from CRCs may be attributed to the higher salary and professional identity of the CRAs (11). Seen comprehensively from educational background and previous work experience, no clear requirements existed for the access standards of the CRA occupation, hence even a large professional span occurred.

### 3.2 Overview of work

A CRA’s daily work is to monitor the trial implementation in different research sites for one thing, and to organize project materials and write project reports in the companies for another. Therefore, the investigation of their work includes two basic sections: current workloads and travel frequency. Considering the professional characteristics of CRAs that due to the differences of each clinical trial, it is necessary for CRAs to keep updated on corresponding clinical knowledge and clinical trial regulations, we have also included training information in the investigation (12).

According to the survey results (Table 2), each CRA was responsible for 4.5 projects on average at present which were distributed in 5.94 hospitals scattered in 3.44 cities of 2.54 provinces, and each of them visited hospitals for 8.32 days every month. If we take as a reference the standard working hours of 8 h per day for 5 days a week, more than 80% of them worked more hours than the standard ones, with 21.79% of them even working over 10 h a day, showing that it was common for CRAs to work overtime who were burdened with relatively heavy workloads. Before the COVID-19 epidemic, the majority of CRAs went on business trips for 1 to 2 weeks per month. But now 47.63% went on business trips for less than 3 days per month, and some even hardly did it. Seeing that CRAs’ work were still greatly affected by COVID-19, it is speculated that subsequently sponsors or CROs would prefer to select or give priority to research sites that are located nearby or can be monitored remotely.

**Table 2:** Workloads, travel frequency, and training of CRAs.

Classification	Number (N)	Proportion (%)
Working hours per week		
Below 40 h	74	18.45
40–50 h	218	54.36
50–60 h	86	21.45
Above 60 h	23	5.74
Business trip days per month (Before COVID-19)		
3 days below	102	25.44
3–7 days	90	22.44
7–14 days	111	27.68
14–21 days	45	11.22
21 days above	6	1.50
Join CRAs after COVID-19	47	11.72
Business trip days per month (After COVID-19)		
3 days below	191	47.63
3–7 days	127	31.67
7–14 days	63	15.71
14–21 days	19	4.74
21 days above	1	0.25
Sources of training (multiple choices)		
Employer	367	91.52
Hospitals	55	13.72
Industry society	104	25.94
Self-study	277	69.08
Frequency of training		
Weekly	102	25.44
Monthly	196	48.88
Quarterly	34	8.48
Annually	31	7.73
Hold on demand	38	9.48
Whether willing to take trainings during personal break		
Unwilling	103	25.69
Willing	298	74.31

Because continuous training is a must for CRAs due to the nature of their work, 74.31% of them were willing to use their break time to study, relying mainly on self-study and company training, with 48.88% receiving training every month. As a whole, most CRAs were very willing to enrich their professional knowledge and improve their professional skills through multiple channels. However, it is also noted that the training organized by hospitals accounts for the lowest proportion, while the reality is that different hospitals had different management requirements and implemented different Standard Operating Procedures (SOP), and hospitals should hold training for CRAs or sponsors to explain the management process, project material list, and monitoring requirements, etc.

### 3.3 Satisfaction with institution sites

The clinical trials are initiated by investigators from research sites, with management departments supervising the whole process of the trial, and at the same time, the Ethics Committee acting as an independent third party to protect the rights and interests of subjects. CRAs go to various sites to monitor the trials and finish their main work in hospitals. Therefore, it is necessary to evaluate their satisfaction with the management and implementation of clinical trials in hospitals so as to reflect the problems encountered in monitoring (13). With four satisfaction grades (very satisfied, satisfied, dissatisfied and very dissatisfied), the respondents were invited to evaluate their satisfaction with investigators, institution sites and ethics committees.

As conductors of trials, investigator’ professionalism and cooperation are directly linked to the quality of clinical trials (Table 3). The evaluation of investigators was divided into four dimensions including their GCP awareness, cooperation degree, familiarity with the protocol and understanding of the management process of clinical trials in hospitals. In terms of GCP awareness, 44.39% of CRAs were dissatisfied, and 11.47% being very dissatisfied. Despite a slight lack of awareness of GCP, 66.33% of CRAs thought that investigators were cooperative. More than half of CRAs held a negative attitude toward investigators’ familiarity with the protocol, thinking that the investigators were not familiar with the protocol or not as familiar as they expected. 60.60% of CRAs regarded that researchers were not familiar with the management process of clinical trials in hospitals, reflecting insufficient training of relevant management rules for investigators provided by hospitals.

**Table 3:** CRAs’ satisfaction with investigators.

Classification	Satisfaction degree	Number (N)	Proportion (%)
GCP awareness	Very satisfied	39	9.73
	Satisfied	138	34.41
	Dissatisfied	178	44.39
	Very dissatisfied	46	11.47
Cooperation in clinical trials	Very satisfied	77	19.20
	Satisfied	189	47.13
	Dissatisfied	119	29.68
	Very dissatisfied	16	3.99
Familiarity with the protocol	Very satisfied	43	10.72
	Satisfied	134	33.42
	Dissatisfied	167	41.65
	Very dissatisfied	57	14.21
Understanding of the management requirements of clinical trials in hospitals	Very satisfied	32	7.98
	Satisfied	126	31.42
	Dissatisfied	164	40.90
	Very dissatisfied	79	19.70

As management departments, the institutional research office are responsible for the project establishment, contract signing, trial drug/device management and project conclusion (Table 4). Also, as the main external communication departments, the onus is on institutional offices to elaborate on the management process of project operation, materials for project establishment and contact information of the institution so as to facilitate CRAs’ communication. As to the feedback from CRAs, 63.34% thought that the management of institutional offices was transparent and satisfactory. Two-thirds of the CRAs were satisfied with the work efficiency of institutions’ management staffs. However, the proportions of the people who were satisfied and dissatisfied were very close in terms of the evaluation of auxiliary departments, indicating much room for auxiliary departments to improve their services.

**Table 4:** CRAs’ satisfaction with institutional research office.

Classification	Satisfaction degree	Number (N)	Proportion (%)
Management transparency	Very satisfied	91	22.69
	Satisfied	163	40.65
	Dissatisfied	122	30.42
	Very dissatisfied	25	6.24
Management staffs’ efficiency	Very satisfied	70	17.46
	Satisfied	171	42.64
	Dissatisfied	134	33.42
	Very dissatisfied	26	6.48
Cooperation of auxiliary departments	Very satisfied	56	13.97
	Satisfied	146	36.41
	Dissatisfied	137	34.16
	Very dissatisfied	62	15.46

The Ethics Committee generally holds regular meetings to review clinical trials to ensure the scientific operation of projects as well as the rights and interests of subjects; and it keeps follow-up reviews in the course of project operation (Table 5). 80.30% of the ethics committees that the CRAs had contacted with held one meeting per month, the frequency of which could meet the needs of the project schedule. More than 80% of the CRAs accepted the opinions given by the ethics committees to the project.

**Table 5:** CRAs’ satisfaction with ethics committees.

Classification	Satisfaction degree	Number (N)	Proportion (%)
Frequency of meetings	Twice a month	52	12.97
	Once a month	322	80.30
	Once 2 months	6	1.49
	Once a quarter	4	1.00
	Hold on demand	17	4.24
Whether the frequency meets the project demands	Satisfy	256	63.84
	Dissatisfied	145	36.16
Ethical opinions	Very satisfied	154	38.40
	Satisfied	167	41.65
	Dissatisfied	70	17.46
	Very dissatisfied	10	2.49

### 3.4 Career satisfaction

This part of the survey aimed to learn about the CRAs’ current satisfaction with the work and their subsequent career planning. Job satisfaction is related to employee retention rate, while low satisfaction leads to resignation intention and thus affects the rate. As Table 6 shows, only 49.63% of them were satisfied with their current salary. According to the survey of job profiles mentioned above, most CRAs bore relatively large workloads but received unsatisfactory salary, with 12.47% of them even very dissatisfied with their current salary.

**Table 6:** CRAs’ career satisfaction.

Classification	Number (N)	Proportion (%)
Current salary		
Very satisfied	5	1.25
Satisfied	194	48.38
Dissatisfied	152	37.90
Very dissatisfied	50	12.47
Promotion channels		
Very satisfied	52	12.97
Satisfied	279	69.58
Dissatisfied	59	14.71
Very dissatisfied	11	2.74
Training arranged by the company		
Very satisfied	76	18.95
Satisfied	239	59.60
Dissatisfied	76	18.95
Very dissatisfied	10	2.50
Opinions on the development of the industry		
Optimistic	346	86.28
Pessimistic	37	9.23
No idea	18	4.49
Follow-up plan		
Continue to work as a CRA for promotion	348	86.78
Switch to other positions related to clinical trials	42	10.48
Switch to other industries unrelated to clinical trials	11	2.74
Aspects focused on when hunting for a job (multiple choices)		
Salary	370	92.27
Company project resources	330	82.29
Working atmospheres	307	76.56
Promotion channels	264	65.84
Internal management, training, etc.	246	61.35
Office location	211	52.62
Frequency of business trips	179	44.64
Reputation of the company	136	33.92
Others	6	1.50

86.28% of CRAs were optimistic about the future development of their careers, and 86.78% were willing to continue their careers as CRAs. If they chose to change their employers, the three aspects that would be paid most attention are as follows: salary accounting for the largest proportion of 92.27%, and company project resources as well as working atmospheres for the rest. Hence, enterprises could consider salary as the most favorable competitive advantage when recruiting CRAs in the future (14). Company project resources were the second factor that CRAs took into consideration, which manifested that they were more willing to choose companies with sufficient high-quality projects in order to realize the improvement of their own abilities through the training at work. The proportion of working atmospheres, as the third factor, was about 10 percentage higher than that of promotion channels, which showed that CRAs were, compared with the promotion, more willing to choose a pleasant working environment with friendly colleague relationships and easygoing leaders. As supplements to the aforementioned factors of salary and working atmospheres, other additional factors that respondents offered included welfare, insurances and the quality of supervisors.

### 3.5 Differential analysis of the satisfaction

Furthermore, we used the one-way ANOVA to compare satisfaction scores with different educational backgrounds, ages, and incomes. According to the Central Limit Theorem, data from large samples (*n* > 30) can be considered approximately normally distributed. The sample size in this study is much greater than 30, so it meets the principle of normal distribution required for analysis of variance. After the Levene test for homogeneity of variances, the *p*-values for all groups are greater than 0.05, indicating that the homogeneity of variances is satisfied, and a one-way analysis of variance can be conducted.

The results show that there was no difference in the satisfaction to investigators, institutional research office and ethics committees, but there were significant differences in the career (*p* < 0.05), suggests a significant educational impact on satisfaction for this group (Table 7). And Table 8 provides a comprehensive analysis of satisfaction scores in different age among CRAs. Our results indicates that there is no significant variation in investigators, institutional research office, ethics committees and career satisfaction scores attributable to age, suggesting a more uniform perception across different age groups. Similarly, we have analyzed the correlation between annual income brackets and satisfaction scores for CRAs (Table 9), the result presents that the different income CRAs have no difference in satisfaction, reminder that income is not the importance factor the overall contentment of CRAs within the industry.

**Table 7:** Differences in satisfaction among CRAs with different educational backgrounds.

Variable	Education level	*N*	Score	*F*	*p*
Investigators	Above Master	67	9.96 ± 2.86	1.916	0.149
	Bachelor	318	10.12 ± 2.64		
	Junior college or below	22	9.85 ± 2.87		
Institutional Research Office	Above Master	67	8.02 ± 2.13	1.614	0.200
	Bachelor	318	7.64 ± 2.03		
	Junior college or below	22	8.08 ± 2.16		
Ethics Committees	Above Master	67	6.57 ± 1.17	2.888	0.057
	Bachelor	318	6.84 ± 1.27		
	Junior college or below	22	6.49 ± 1.13		
Career satisfaction	Above Master	67	7.37 ± 1.02	3.038	0.049
	Bachelor	318	7.17 ± 1.01		
	Junior college or below	22	7.43 ± 0.98		

**Table 8:** Differences in satisfaction among CRAs with different age.

Variable	Age	*N*	Score	*F*	*p*
Investigators	21–25	96	9.97 ± 2.87	1.309	0.271
	26–30	239	9.55 ± 2.89		
	>31	73	10.09 ± 2.81		
Institutional Research Office	21–25	96	8.03 ± 2.13	1.547	0.214
	26–30	239	7.70 ± 1.84		
	>31	73	8.15 ± 2.25		
Ethics Committees	21–25	96	6.57 ± 1.16	0.542	0.582
	26–30	239	6.47 ± 1.29		
	>31	73	6.62 ± 1.14		
Career satisfaction	21–25	96	7.37 ± 1.02	0.228	0.796
	26–30	239	7.41 ± 1.04		
	>31	73	7.34 ± 0.94		

**Table 9:** Differences in satisfaction among CRAs with different income.

Variable	Annual income	*N*	Score	*F*	*p*
Investigators	Below 100,000	83	9.96 ± 2.86	1.821	0.163
	100,000–200,000	226	10.35 ± 3.03		
	Above 300,000	100	10.00 ± 2.81		
Institutional Research Office	Below 100,000	83	8.02 ± 2.13	0.575	0.563
	100,000–200,000	226	8.24 ± 2.16		
	Above 300,000	100	7.99 ± 2.14		
Ethics Committees	Below 100,000	83	6.57 ± 1.17	0.554	0.575
	100,000–200,000	226	6.65 ± 1.33		
	Above 300,000	100	6.51 ± 1.08		
Career satisfaction	Below 100,000	83	7.37 ± 1.02	1.027	0.359
	100,000–200,000	226	7.23 ± 1.14		
	Above 300,000	100	7.39 ± 0.95		

### 3.6 Suggestions

**Figure 1:**
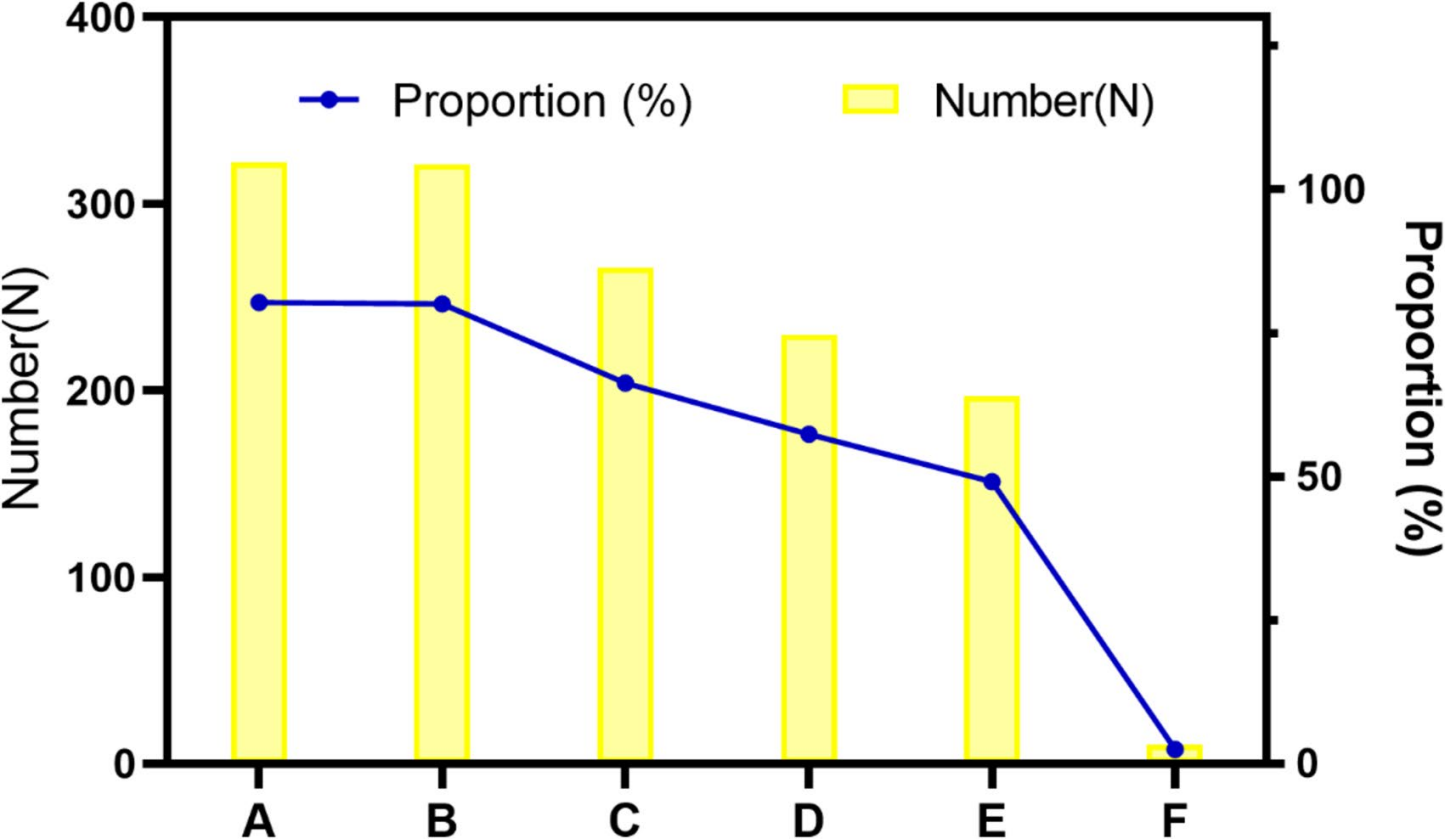
Stacked bar chart of CRAs’ suggestions for the industry. A: Establish unified clinical trial application materials nationwide. B: Promote mutual recognition of ethical results, C: Promote mutual recognition of ethical results, D: Establish a unified CRA grade evaluation, E: Organize national certification examinations, F: Others.

In the last part of the research, the CRAs were invited to make some suggestions to the industry. According to our usual work experience, we put forward five suggestions and asked the CRAs to evaluate whether they were applicable. In addition, we consulted the respondents about their personal suggestions on the subsequent improvement of the industry so as to solve the difficulties encountered in their current work (Figure 1).

It was suggested by more than 80% of the CRAs that it was necessary to establish nationally unified clinical trial application materials and implement a mutual ethical recognition system. These two aspects are the most time- and energy-consuming parts in the early stage of clinical trials. Firstly, most hospitals formulate inconsistent SOPs based on their own operation and management requirements. The repeated preparation of project application materials according to the requirements of different hospitals often wastes much of the energy of CRAs. Secondly, as for the ethical recognition system, most ethics committees hold one meeting per month in hospitals at present, as is mentioned above. Multi-site clinical trial projects need to pass the ethical approvals of all research sites involved. Because sponsors are concerned that the ethics committee of one site raises some suggestions on revision, they often wait for the ethical approvals by all sites before implementing the trial. The whole process is so time-consuming that it is necessary to implement a mutual recognition system of ethical results.

66.33% of CRAs thought it necessary to organize national CRA training. At present, the National Medical Products Administration website offers no training programs for CRAs. Most CRAs are trained through their companies’ internal training programs with different contents and difficulty levels, which spawn on uneven training results. The high turnover of CRAs brings about repeated training of similar contents across different companies, resulting in wastes of resources. In the future, it is hoped that unified CRA trainings will be carried out nationwide. 57.36% of CRAs suggested that a national unified CRAs grade evaluation be established while only 49.13% thought it necessary to organize a national certification examination. It is speculated that the CRAs do not want to raise the current access threshold but hope to establish a unified and peer-recognized grade evaluation, which may be helpful to obtain recognition of their abilities when they switch to new positions or new companies.

The suggestions put forward by the CRAs were as follows: (1) establishing a national CRA blacklist; (2) ranking institutions nationwide; (3) improving CRC quality; (4) improving research institution’s electronic information system; (5) establishing remote monitoring channels; (6) enhancing recognition of the CRA occupation. Firstly, according to the aforementioned results, the average number of projects that each CRA took over was five times that completed. Because a large part of projects are taken over halfway, poor monitoring quality of the succeeded projects may occur, requiring the successors to constantly improve and make up for the problems left by their predecessors. When a national CRA blacklist is established, potential successors can initially evaluate the project quality and even refuse to take over the blacklisted CRA’s projects. This method can boost the improvement of CRAs’ own quality to a certain extent. Secondly, the national ranking list of research institutions is suggested to be set up. With the list as a reference, it is convenient for CRAs to screen research sites without the need to investigate one by one. And in turn, it can promote the improvement of the management quality of the institution itself. In addition, a CRC plays the role of a housekeeper in clinical trial projects and has such responsibilities as collation of clinical trial materials, communication with subjects, and arrangement of researchers’ time, etc. Whether the housekeeper is serious or responsible or not affects the quality and progress of the trial, hence appealing to improve the quality of CRCs (15, 16). Further, it is imperative for “institutions to improve information systems so as to construct remote monitoring channels” during the COVID-19 epidemic at the moment. The epidemic forced CRAs not to travel as frequently and enter hospitals as freely as before, resulting in a reduction in the frequency of monitoring. If the hospital can establish a clinical trial management system, which provides remote monitoring and eliminates space limitation, it will not only improve monitoring efficiency but also effectively reduce the operating costs of the industry (17). The last one is the suggestions to improve the professional recognition of CRAs. The reasons for this can be concluded as follows: CRAs from sponsors or CROs are sent to hospitals to participate in clinical trials, with few hospitals preparing office space for them. Moreover, non-hospital staff are in a relatively weak position when coordinating and communicating with relevant departments of hospitals, and they are prone to feel psychologically isolated, hence their lack of sense of belonging and professional identity.

## 4 Discussion

Through this investigation, we have a preliminary understanding of the occupational situation of CRAs. The majority of this increasingly young group are women and hold bachelor’s degrees, with educational background and previous work experience related to pharmaceutical industries. However, some CRAs originally engaged in completely irrelevant occupations, or have neither related educational background nor work experience, indicating a low access threshold for working as CRAs. Other countries have established CRA certification mechanisms, such as Association of Clinical Research Professionals (ACRP) (18). Presently, since practitioners in the medical industry, such as doctors, nurses, pharmacists, laboratory technologists, etc., have exacting qualification requirements, only after studying relevant majors and passing national qualification examinations, can they be qualified for corresponding positions. As for CRAs who are also part of the medical industry, firstly, there is a scarcity of educational resources shared by them. At present, almost no universities or vocational schools in China offer clinical trial majors except that a tiny number of medical schools arrange clinical trial-related courses in some medical majors. For example, the “clinical trial training” is a course set in the clinical pharmacy major of China Pharmaceutical University. Secondly, no CRA qualification examination or corresponding registration requirements are listed in the current planning of medical human resources. CRAs, as highly specialized practitioners, should not only have a basic command of medical knowledge but also be familiar with clinical trial regulations and requirements. It is necessary to manage the CRAs as a whole, through establishing applicable professional standards and talent evaluation system so as to guide the positive development of the industry. In summary, the lack of talent reserve and imperfect management lead to different quality of employees and even to the emergence of employees from non-medical related majors.

It is estimated that the relative maturity in drug clinical trials leads to drug CRAs up to about three quarters of all CRAs. The laws and regulations governing clinical trials originated from drug clinical trials. Not until 2016, did China have its exclusive “Standard for Quality Management of Medical Device Clinical Trials” for medical devices. Therefore, the development of drug clinical trials was earlier and more standardized than that of medical device in the whole industry, leading to difference in the number of CRAs engaging in drug trials and medical device trials. Surprisingly, we find in this survey that CRAs also engage in scientific research projects that are non-registered clinical researches. It is analyzed that the standardization of clinical trials steals the limelight, especially registration-oriented trials carried out in accordance with GCP specifications. With GCP’s mature management system as a reference, other clinical studies have begun to add in CRAs and other positions to monitor the research quality, such as clinical studies initiated by government or relevant research societies (19, 20). It can be predicted that there will be a rise of CRAs in non-registered clinical research projects in the future.

Research shows that the annual income of high-income people in China was about 80,000 RMB in 2020 (21). According to the CRAs in the survey, 80% of them have an annual income of more than 100,000 yuan, belonging to high-income groups. Our result shows the 95.01% CRAs held bachelor’s degrees or above, corresponding to the relationship between education and income, i.e., higher education leads to higher income (22, 23). As the clinical trial business is booming now, the demand for CRAs is also increasing, in line with the law of supply and demand, the salaries of CRAs have marked increases (14). Salary also serves as the driving force for an increasing number of people, especially fresh college graduates, to choose to work as CRAs. At the same time, compensation plays the most important role in navigating CRAs’ future career planning. It is similar to the research results of CRA groups abroad, the CRA survey results in Canada show that low salary, unmanageable workload, lack of career advancement and professional develop and cooperation from medical teams are the main factors for resignation, with low salary being the most important factor (6).

CRA’s responsibility is to monitor the implementation of clinical trials in research sites to ensure the quality of the trials, the impediments to which can be summarized from three aspects: hospitals, enterprises and CRAs themselves. In terms of hospitals, investigators’ awareness of GCP remains to be improved and efforts dedicated to clinical trials to be reinforced, and CRAs also need to be provided with an office environment with satisfactory hardware. With respect to enterprises, the burdensome workload for some CRAs and the insufficient training for the project gives rise to undesirable monitoring quality. As for CRAs themselves, they are lacking in such professional knowledge as clinics, GCP, and regulations, etc.

In surveying CRAs’ satisfaction with institutions, more than half of them think that investigators’ awareness of GCP remains to be enhanced. Distinctions should be drawn between the more universalized clinical trials and the more individualized routine clinical diagnoses and treatments, which requires the training and increase of investigators’ GCP awareness. In this study, we did not conduct any research into the frequency of training for investigators in hospitals, but the feedback from CRAs and other research results revealed unsatisfactory training results (8). The training organized by hospitals that CRAs participate in accounts for the lowest proportion among all kinds of training sources. It is suggested that the training in hospitals be open to CRAs at the same time so that both investigators and CRAs can know about the management process of clinical trials. Moreover, since most of the training that CRAs receive in enterprises and self-study materials are from the perspective of the sponsor while the GCP training held by hospitals are from the standpoints of investigators and management departments, the role changing empowers CRAs to have a deeper understanding of the connotation of GCP and think from multiple angles during monitoring. In addition, hospitals’ project training can expound on more detailed clinical knowledge, serving as a supplement to the shortcomings of clinical knowledge of CRAs without clinical background.

CRAs’ high turnover due to high frequency of job-switch is also thought-provoking to us. Frequent replacement of CRAs can affect the quality of projects that might be called into question whether the project handover between former and replacing associates is comprehensive and whether the successors have a clear understanding of the information and materials of the projects and subjects (1). For enterprises, other invisible costs brought by the mobility of employees, such as recruitment and training costs, have caused certain losses to enterprises. As far as CRAs themselves are concerned, most are still enthusiastic about this industry and are willing to pay effort to acquire relevant knowledge while continuing their careers in this industry. To sum up, enterprises are suggested to focus on the following aspects so as to ensure the retention of their employees: (1) balancing the workloads of CRAs and avoiding excessive overtime; (2) assigning them to research sites nearby to reduce the frequency of business trips; (3) offering compensation and promotion channels matched with workloads and personal abilities; (4) arranging a possibly large amount of relevant training to provide employees with channels for growth (6).

According to the suggestions offered by the CRAs, they are more concerned about the convenience of project implementation, such as mutual recognition system of ethical results, construction of hospital information system, etc., all of which are inseparable from the overall improvement of the entire industry. Ethical approval can be given by endorsing the results of either the leading institution or a designated hospital. Compared with the current review mode, this practice requires less reviews and hence demands higher quality of ethical review-that is, it is necessary to strengthen the construction of ethics committees in hospitals. A full-fledged information system on the hospital side would allow CRAs to work from remote locations, yet it entails perfect collaboration between the HIS (Hospital Information System, HIS), LIS (Laboratory Information Management System, LIS) and PACS (Picture Archiving and Communication Systems, PACS) on the hospital side and the EDC (Electronic Data Capture System, EDC) on the enterprise side. This requires a strong information system on the hospital side to accurately capture the information about the participating subjects without leaking the information of other patients.

## 5 Conclusion

Overall, this paper is a preliminary exploration on the CRA occupation. Attention needs to be paid to the needs and opinions of the increasing CRA practitioners who join the continuously thriving pharmaceutical industry. The effective performance of CRAs is instrumental in effectively ensuring the quality of clinical trials and the safety of drugs to be marketed (13). The improvement of the occupational education of CRAs can not only ensure the quality of clinical trials but also resolve the shortage of human resources to some extent (10). Since there is still a gap between China’s CRA development and that of developed countries at present, the subsequent development and quality improvement of China’s CRA is closely linked to the overall improvement of other relevant practitioners in clinical trials, including the improvement of the management abilities of institutions, the quality construction of the ethics committees, and the consolidation of investigators’ GCP awareness (15, 16). In addition, as employers, enterprises should also provide matching compensation and benefits to CRAs and further enhance their professional identity, sense of belonging and career enthusiasm so as to generate more CRAs with high quality.

This survey is the preliminary exploration of the occupational situation of CRAs in China. We have knowed the specific difficulties of CRAs in clinical trial supervision, and put forward improvement suggestions, which provides support for improving the professional status of CRAs, thereby further improving the quality of clinical trials. However, due to resource and time constraints during the COVID-19 epidemic, we were unable to receive a large enough sample size and the sample was not representative enough. Likewise, although we have tested the reliability and validity of the questionnaire and the results are good, as non probabilist sample, there is a conflict between reliability and validity, may leading a possible bias in the interpretation of the results. While the survey scope of this study basically covers the whole country, and we have a preliminary understanding of the overall occupational status of CRAs. It’s also provides us with a good foundation for further expanding the sample size and conducting in-depth investigations over a long enough period to understand this group.

## Data Availability

The original contributions presented in the study are included in the article/Supplementary material, further inquiries can be directed to the corresponding author.

## Footnotes

^1^ https://www.wjx.cn
